# Appointments in Medical Colleges

**Published:** 1853-10

**Authors:** 


### APPOINTMENTS IN MEDICAL COLLEGES.

Dr. SOCRATES MAUPIN has been appointed to the Chair of Chemistry in the University of Virginia, vacated by the resignation of Dr. J. LAWRENCE SMITH, who has settled permanently in Louisville.

Dr. E. H. PARKER, Editor of the *New Hampshire Juornal of Medicine*, has been appointed Professor of Physiology and Pathology in the New York Medical College.

The following appointments have been made in the Medical College of Ohio: Dr. G W. BAYLESS, Professor of Anatomy; Dr. ASBURY EVANS, of Covington, Ky., Surgery; Dr. CHARLES W. WRIGHT, Chemistry; Dr. N. T. MARSHALL, Midwifery; Dr. SAMUEL G. ARMOR, Physiology and Pathology.

In the Medical Department of Yale College, Professor SILLIMAN, Sen., having resigned the chair of Chemistry, and Dr. ELI IVES that of Materia Medica, Dr. HENRY BRONSON has been appointed to the latter, and Dr. BENJAMIN SILLIMAN, Jr., succeeds his father in the former. The last appointment, as we have heretofore said, makes no change in the programme of exercises in the University of Louisville for the coming session. Prof. SILLIMAN is still in the Chemical chair. His health, which suffered from too severe mental application during the hot weather in August, is, we are happy to say, entirely restored, and he expects to be in Louisville early in October to engage in the preliminary course.

